# Sustainable Water Resource Management of Regulated Rivers under Uncertain Inflow Conditions Using a Noisy Genetic Algorithm

**DOI:** 10.3390/ijerph16050868

**Published:** 2019-03-09

**Authors:** Chunxue Yu, Xinan Yin, Zhifeng Yang, Zhi Dang

**Affiliations:** 1 Research Center for Eco-environmental Engineering, Dongguan University of Technology, No 1 Daxue Street, Songshan Lake, Dongguan 523808, China; yuchunxue121@163.com; 2 School of Environment and Energy, South China University of Technology, University Town, Guangzhou 510006, China; chzdang@scut.edu.cn; 3 State Key Laboratory of Water Environmental Simulation, School of Environment, Beijing Normal University, No 19 Xinjiekouwai Street, Beijing 100875, China; yinxinan@bnu.edu.cn

**Keywords:** environmental flow, reservoir operation, stochastic inflow, noisy genetic algorithm

## Abstract

Ecofriendly reservoir operation is an important tool for sustainable water resource management in regulated rivers. Optimization of reservoir operation is potentially affected by the stochastic characteristics of inflows. However, inflow stochastics are not widely incorporated in ecofriendly reservoir operation optimization. The reasons might be that computational cost and unsatisfactory performance are two key issues for reservoir operation under uncertainty inflows, since traditional simulation methods are usually needed to evaluate over many realizations and the results vary between different realizations. To solve this problem, a noisy genetic algorithm (NGA) is adopted in this study. The NGA uses an improved type of fitness function called sampling fitness function to reduce the noise of fitness assessment. Meanwhile, the Monte Carlo method, which is a commonly used approach to handle the stochastic problem, is also adopted here to compare the effectiveness of the NGA. Degree of hydrologic alteration and water supply reliability, are used to indicate satisfaction of environmental flow requirements and human needs. Using the Tanghe Reservoir in China as an example, the results of this study showed that the NGA can be a useful tool for ecofriendly reservoir operation under stochastic inflow conditions. Compared with the Monte Carlo method, the NGA reduces ~90% of the computational time and obtains higher water supply reliability in the optimization.

## 1. Introduction

There are about 58,000 large dams in regulated rivers all over the world, and ~3700 new dams are proposed in developing economies [1,2]. Although dams provide water resources for irrigation, municipalities, and hydropower production, they also have caused serious disruption to aquatic environments [3]. Recent researches have paid increasing attention to how dams could be operated for sustainable water resource management [4]. Most of the studies suggest that it should operate the reservoir in an ecofriendly manner to meet both human and riverine ecosystem requirements [5,6,7]. Ecofriendly reservoir operation is a useful way to achieve this goal, which determines reservoir operation rules ensure not only the human interest but also the protection of downstream riven ecosystem health. Different from traditional operation only satisfying the utilizable request, ecofriendly reservoir operation also aims to protect the flow regime for downstream ecosystem by incorporating different environmental flow (e-flow) release policies [8,9,10,11,12,13,14,15]. Identifying effective ecofriendly reservoir operating rules will maximize the beneficial use of these projects.

Identification of optimal ecofriendly reservoir operating rules is not easy, due to the various uncertain conditions in reservoir operation [16,17]. The stochastic nature of inflow is one of the unavoidable uncertainties [18,19,20]. Existing studies have usually ignored this problem and used a deterministic inflow (such as historical inflows) as an input for optimization of ecofriendly reservoir operations [8,9,10,11,12,13,14,15]. However, reservoir inflow depends entirely on rainfall in a river basin. Especially in areas characterized by randomly distributed seasonal rainfall, reservoir inflow is a stochastic variable [21,22,23]. Meanwhile, there is a low probability of reoccurrence of historical inflows and historical inflows (like 1950–1969) are very different from the future inflows (like 2020–2039) due to the influences of anthropogenic activity and climate change [24,25]. The proposed operating rules based on historical inflows might not competently balance the diverse water demands of human and the river ecosystem in the future. Hence, it is useful to consider the impacts of uncertainties in reservoir inflows on the optimization of ecofriendly reservoir operations. According to the previous studies, several methods are proved to be useful in handling the stochastic characteristics of inflows, including stochastic dynamic programming (SDP), stochastic linear programming (SLP), Monte Carlo method, and so on [16,18]. However, these methods are often required to carry out many times and the results vary between different realizations. Computational cost and performance usually cannot be satisfied at the same time.

To deal with the uncertain inflow conditions in ecofriendly reservoir operation, a noisy genetic algorithm (NGA), which is especially effective in dealing with stochastic problems, could be an alternative approach [26]. Different from the tradition simulation methods, the NGA has been verified that it can run well without sampling plenty of realizations in different optimizations [27,28]. NGA is a standard genetic algorithm which can perform well in a noisy environment. In practice, a noisy environment (e.g., the stochastic nature of inflows) is commonly seen in actual problems (e.g., optimization of reservoir operation). A sampling fitness function is designed to be adopted in the NGA to lessen the noise of fitness assessment. Smalley at al. [26] effectively coupled the NGA with a transport model, and found that it could identify the highly reliable designs for the situ bioremediation system with a small amount of samples. Chan Hilton and Culver [29] compared the performance of basic GA, noisy GA, and robust GA in dealing with the uncertainty of aquifer parameters, and the results showed that the noisy GA could obtain higher reliability designs than others. Wu et al. [30] attempted to solve the sampling network design problems based on the sampling of NGA under uncertainty. Singh and Minsker [31] and Yan and Minsker [32] proved that NGA performed well in optimizing the groundwater remediation designs. Researcher used a NGA to optimize stochastic reservoir operation and proved that a NGA can obtain better results than other methods [33]. However, a NGA has not been applied in the optimization of stochastic ecofriendly reservoir operations.

This study explores the effectiveness of a NGA in ecofriendly reservoir operation under different stochastic inflow conditions. To assess its impact on stochastic ecofriendly reservoir operation, the performances of the NGA and the Monte Carlo method, which is another common approach to stochastic problems, are compared. The satisfaction of human needs is reflected by water supply reliability and the influence on environmental flow requirements is characterized as the alteration of a set of indicators based on the range of variability approach (RVA) [34]. As an illustrative example—a reservoir in China—was selected to evaluate the effectiveness of the two methods. 

## 2. Methods

In this study, to incorporate the effect of stochastic inflows on ecofriendly reservoir operation, a Monte Carlo combined standard genetic algorithm (GA) and a noisy genetic algorithm (NGA) were developed to optimize reservoir operating rules. The reservoir operating rule curves (RORCs) was employed to guide the reservoir operation [35,36]. We used the water supply reliability to describe the satisfaction of human need, and the degree of flow regime alteration to reflect the satisfaction of ecosystem requirement. To get diverse reservoir operating schemes, the environmental flow (e-flow) releases were guided by three commonly used policies. We intended to use different policies to capture different e-flow requirements. For each policy, we carried out the optimization independently with the objective of maximum water supply reliability, *R* (the objective of actual reservoir operation). 

### 2.1. E-Flow Allocation

#### E-Flow Policies

This study considers three commonly used e-flow allocation policies shown in Table 1. E-flow is described as the flows immediately released downstream of the reservoir to protect the river flow regime. It consists of either or both a required release and uncontrolled spills. Actually, the river managers are only able to obtain yesterday’s reservoir inflow data. So the optimization in this study assumes that the e-flow for the next day is determined by averaged inflows of the prior day from initially available daily data. The three policies are described as follows.

Policy 1: Fraction of Inflow (FOI)

A fraction of the reservoir inflow is released as e-flows. The fraction is defined from 0.1 (FOI = 0.1) to 0.9 (FOI = 0.9), thus this policy includes nine scenarios.

Policy 2: Flow Components (FC)

According to the Tennant method [37], this policy uses 10% of average daily flow (ADF) as e-flow during the dry season and 30% during the wet season e-flow. This policy also tries to deliver occasional high-flow releases [38], which are greater than the 75th percentile for all flows. The quantity of high-flow events in a given scenario is decided beforehand. Once the practice is done, no further high-flow event will occur. The scenario must, at least, include three high-flow events and then next scenario will add three more; that is, scenario 1 has three high-flow events, scenario 2 has six, and so on.

Policy 3: Four-period release approach (FP)

In the FP approach proposed by Yin et al. [11], the e-flow requirement of four basic flow periods in a year is as follows. (i) Floods: The 1.5-year flood is equal to an estimate of the bankfull discharge (BD), and bd is released in a flood period for e-flow. (ii) Base flows: This period uses 10% of ADF as e-flow during the dry season and 30% of ADF during the wet season. (iii) Extreme low flows: e-flow equals to the reservoir inflow. (iv) High-flow pulses: all high-flow are released to the downstream.

The range of variability approach (RVA), which is one of the most commonly used approaches for flow regime alteration assessment, is used in this study [34]. Magnitude, timing, frequency, duration, and rate of change are the indicators in the RVA (Table 2). The degree of alteration, *D_m_*, measures the *m*_th_ hydrological indicator’s deviation of the altered flow regime from the natural one:which is defined as follow

(1) $$D_{m}=\left|\frac{N_{o,m}-N_{e,m}}{N_{e,m}}\right|\times 100\%$$

where *N_o_*_,*m*_ is the observed number of the *m*_th_ hydrological indicator’s value within its RVA target range in postimpact years; *N_e_*_,*m*_ is expected number of the *m*_th_ hydrological indicator’s value within its RVA target range in postimpact years. The average of these hydrological indicators’ alteration is used to represent the overall influence on a river:

(2) $$D=\frac{1}{H}\sum_{m=1}^{H}D_{m}$$

where *D* is the total flow regime alteration, and *H* is the number of these hydrological indicators. 

According to RVA, the degree of flow regime alteration can be classified into three-levels: low, representing values of *D* between 0 and 33%; moderate, representing *D* between 33% and 67%; and high, representing *D* between 67% and 100%.

### 2.2. Reservoir Operation Model

**Figure 1:**
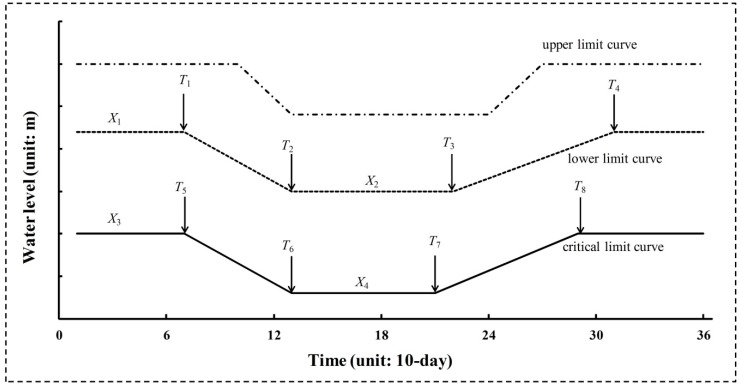
Typical reservoir operating rule curves.

Reservoir operating rule curves (RORCs) have always been used to direct reservoir operation. A typical RORCs graph is shown in Figure 1. On the basis of three curves (including upper, lower, and critical limit curves), the reservoir is divided into four zones [39,40]. Different zones’ water levels correspond to different water supply reliabilities. The upper limit curve is determined during reservoir design, and represents the reservoir’s flood control function. The upper limit curve remains the same as design in this paper. The lower and critical limit curves represent the high and low storage zones. As shown in Figure 1, *X*_1_ and *X*_2_ are the water levels for the lower limit curve, and *X*_3_ and *X*_4_ are the water levels for the critical limit curve. *T*_1_, *T*_2_, *T*_3_, and *T*_4_ are the initial and ending times of the transition zones for the lower limit curve, and *T*_5_, *T*_6_, *T*_7_, and *T*_8_ for the critical limit curve. 

Water supply for human consumption and the provision of e-flows could be affected by changes in these curves. Ecofriendly reservoir release policies are determined based on water levels for particular limit curves. Different water supply hedging rates will be triggered by water levels in different zones. The α and β hedging rates (0 < α < β <100) are decided based on the experience of reservoir manager.

(1) If the water level rises above the upper limit curve, more water needs to be released to keep the water level less than the upper limit.

(2) If the water level lies between the upper and lower one, it can normally satisfy human water needs and the e-flow requirement for the downstream river. 

(3) If the water level lies between the lower and critical one, the e-flow requirement can be satisfied, but α% of human water supply should be cut.

(4) If the water level reduces below the critical limit curve, the β% of human water supply should be cut. E-flow releases cannot be completely satisfied due to that reservoir water level must stay above the dead storage level. 

To obtain the RORCs (variables of water level *X_i_* (*i* = 1, 2, 3, 4) and time *T_j_* (*j* = 1, 2, …, 8)), the reservoir operation objectives and constraints with the consideration of e-flow allocations are designed as follows. A simple water balance equation (quantizing the change in storage influenced by inflow and outflow) is adopted in the reservoir operation model:

(3) $$S_{i}-S_{i-1}=I_{i}-\left(W_{i}+M_{i}\right)$$

where *S_i_* is the reservoir storage at the end of period *i*, *M_i_* is the mass of water diverted from the reservoir to water supply uses during period *i*, *W_i_* is the water release from the reservoir during period *i*, and *I_i_* is the inflow to the reservoir during period *i* [13].

In each e-flow allocation policy, the objective of reservoir operation is designed to maximize water supply reliability, shown as follows.

(4) R=max(SI)

where *SI* is water supply reliability: *SI* = (actual water supply)/(planned water supply).

*SI* is subject to the following constraints for *X_i_* and *T_j_*.

(5) $$X_{\mathrm{MAX}}>X_{1}>X_{2}$$

(6) $$X_{\mathrm{MAX}}>X_{1}>X_{3}$$

(7) $$X_{2}>X_{4}>X_{\mathrm{MIN}}$$

(8) $$X_{3}>X_{4}>X_{\mathrm{MIN}}$$

(9) $$1\leq T_{1}<T_{2}<T_{3}<T_{4}<36$$

(10) $$1\leq T_{5}<T_{6}<T_{7}<T_{8}<36$$

where *X*_MAX_ is the maximum acceptable reservoir storage level and *X*_Min_ is the minimum admissible storage level. The units of *X* and *T* are m and 10-day, respectively.

### 2.3. Optimization Methods

#### 2.3.1. Monte Carlo Genetic Algorithm

The Monte Carlo Genetic Algorithm means that the simple GA runs multiple times according to the extensive realizations generated by Monte Carlo. For each realization generated by the Monte Carlo method, the optimization of reservoir operation becomes deterministic and is optimized independently by the simple GA. The optimal solutions of multiple realizations are usually averaged as the final optimal solution.

The first step of the Monte Carlo GA is to generate multiple realizations (sets of inflow) based on the Monte Carlo method. Statistically, every realization is equally possible. In this study, we assume that the time series data of the reservoir daily inflow is independent and uniformly distributed, namely *I* = $\bar{I}$ + *σ*, where *I* is the daily inflow, $\bar{I}$ is the average value of daily inflow, and *σ* is a random variable with daily flow mean and standard deviation equaling to zero. The generated *n* realizations of daily inflow based on the averages and standard deviations of historical data will be used as the input of reservoir operation.

In the second step for every realization, the standard GA is used to optimize the reservoir operation and the outputs of *n* realizations are finally averaged as the optimal solution. To obtain the outputs, it needed to initialize population (parameters of RORCs) by GA. The fitness of every individual parameter in the initial population is determined based on the objective function and constraints. The GA uses three operators—selection, crossover, and mutation—to direct the population approaches to the global optimum. The selection operator is designed for choosing better individuals. In this process, the stochastically chosen parameters are compared with each other and then the best individual is selected in the current generation. The crossover and mutation operators are designed to reproduce solutions. In the crossover process, under a specific probability, chosen individuals are coupled together to mate. In the mutation process, the stochastically chosen bits among the new individual outputs are changed under a given probability of mutation.

#### 2.3.2. The Noisy Genetic Algorithm (NGA)

As mentioned previously, a noisy genetic algorithm (NGA) is a standard GA that operates in a noisy, real-world environment [31]. In the optimization of reservoir operation, the noisy environment means the stochastic character of daily inflow. Most components of a NGA are the same as those of a standard GA, while the key difference is the design of the fitness function. For the NGA, it is impossible to obtain the exact value of fitness due to the variability of daily inflows. So the value of fitness is designed to be substituted by an expected fitness value. There are three basic parts of NGAs:

(1) Expected fitness of noisy GA: The expected fitness of an individual’s output for random daily inflows can explore its fitness landscape at several random points in its neighborhood and calculate its average value. The anticipated fitness value of each individual output for stochastic daily inflows possibly can be characterized as an average of several fitness values of the nearby stochastic points. Before the assessment of a fitness function *f* (*x*, *e*) (where *x* is the solution and *e* is the optimization environment), the solution *x* follows the distribution with *δ*, that is *x*→*x* + *δ*. The sampling method of solution *x* is completed according to random disturbance, and each disturbance is independently selected in the neighborhood of disturbance *δ* [41]. Then the objective of the NGA can be transformed to minimize the anticipated value of E(f(x+δ)) over the range of disturbance. After defining a probability density function *p*(*δ*) of disturbance *δ*, the anticipated fitness could be expressed as

(11) $$f\left(x\right)=E\left(f\left(x+\delta \right)\right)=\int_{-\infty }^{\infty }p\left(\delta \right)\cdot f\left(x+\delta \right)d\delta$$

(2) Sampling method. The sampling method is used to disturb solution *x*. The random disturbance is commonly used approach to produce a wide range of random disturbances, *δ*. Other methods, such as Incremental Latin Hypercube and Monte Carlo, have also been adopted for the sampling [42]. Apart from the same parameters in simple GA, the simple size is another parameter special for noisy GA. Sample size is the number of samples specified to calculate the expected fitness of an individual. It has been proved that larger sample sizes do not guarantee better optimal results [43,44]. 

(3) Selection of the final solution. Based on an evaluation of anticipated fitness, the fittest individual solution is not necessarily the best solution. To address this problem, researchers have introduced several possible approaches to find the final solution. In general, there are three possible ways for final solution selection: (I) Optimizing Expected Fitness. (i) Explicit Averaging. A common approach to obtain the anticipated fitness was to evaluate every individual of the final population many times (i.e., 50 times) using random seeds and chose the best one as the final solution [41]. (ii) Implicit Averaging. Increasing the population size can reduce the influence of the noise and, thus, ensure a correct convergence of the algorithm [42]. (II) Multiobjective Approaches. To avoid that the expected function might not sufficient under some circumstances, it is feasible to consider high fitness variance and to search for solutions under different tradeoffs [43]. Branke [44] compared these three approaches and suggested the first one to obtain the final solution selection; we also adopted the same one in the study.

## 3. Case Study

**Figure 2:**
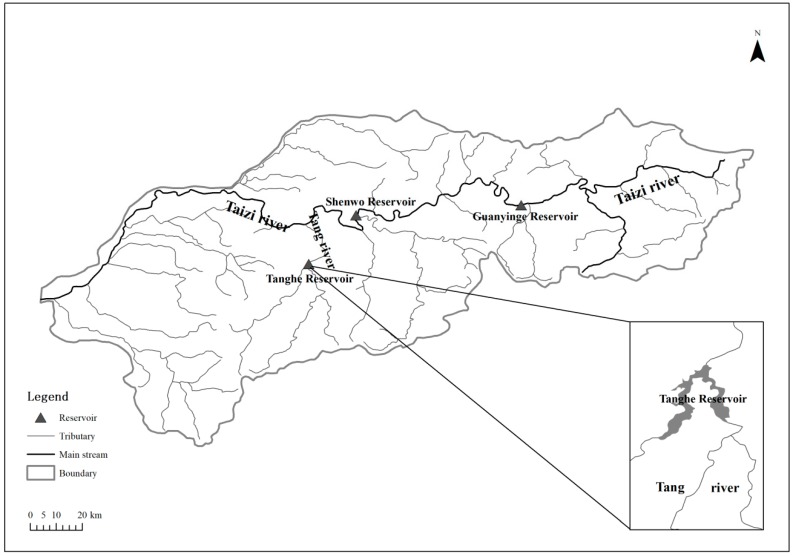
Location of the Tanghe Reservoir in the Taizi River basin.

A single reservoir, the Tanghe Reservoir, is selected as an illustrative example. The Tanghe Reservoir is built on the main section of the Tang River, one of the main tributaries of the the Taizi River (as shown in Figure 2). There are three large reservoirs located in the Taizi River basin—Guanyinge Reservoir, Shenwo Reservoir, and Tanghe Reservoir; there are no reservoirs located upstream of the Tanghe Reservoir. The dam was completed in 1969. The capacity of the reservoir is 707 × 10^6^ m^3^ and the drainage area is 1228 km^2^. Its capability of release is 282 m^3^ s^−1^ and spillway is 2713 m^3^ s^−1^. It is daily operated and has multipurpose. Prior to 1969, there was no water project or large-scale water user in the Tang River, and little water was taken from the upstream river. So the inflow of the Tanghe Gauging Station, recorded from 1950 to 1969, was barely affected by human activities and could be used as natural flow of the reservoir. To simplify the computation, we only considered the operational aim of water supply in this study. Four main water users including Liaoning Chemical Industry Group, Liaoyang Domestic Water Supply Company, Gongchangling Mine Industry Company, and Anshan Domestic Water Supply Company were yearly planned to supply 54.8 × 10^6^ m^3^, 36.5 × 10^6^ m^3^, 18.3 × 10^6^ m^3^, and 73 × 10^6^ m^3^ of water, respectively.

In this study, our reservoir operation model only considers a single-purpose reservoir (i.e., water supply). We ignore the impact of the water use of residents in Tang River due to that it has little influence on flow regime of river. However, although the ecofriendly reservoir operation model only consider the water supply purpose, the optimization results are also appropriate for the reservoir systems including irrigation, navigation and other functions. Only the studied reservoirs like the Tanghe Reservoir, which conserves water in the wet season and consumes water in the dry season.

## 4. Results and Discussion

### 4.1. Analysis of Historical Inflow 

The bd (that is the 1.5-year flood) of Tanghe Reservoir was 42.38 m^3^ s^−1^, ADF was 6.9 m^3^ s^−1^, and the mf was 295.08 m^3^ s^−1^. Based on the Tennant method, there was 10% ADF for the baseflow during the dry season (November to April) was 0.69 m^3^ s^−1^ and 30% ADF for the baseflow during the wet season (May to October) was 2.06 m^3^ s^−1^. There are 25 high-flow events in total of the dry year at Tanghe Reservoir, thus Policy 2 has eight scenarios (from FC = 3 to FC = 24).

**Figure 3:**
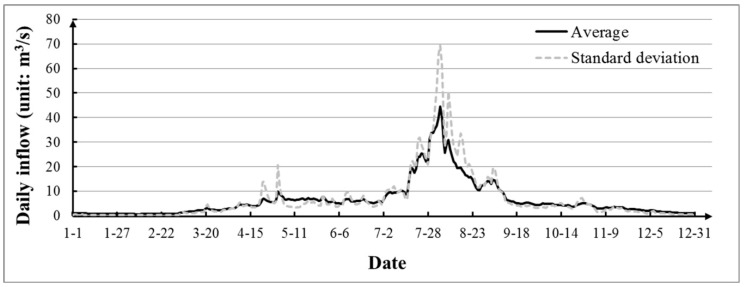
Averages and standard deviations of daily inflow to the Tanghe Reservoir.

The twenty-year (from 1950 to 1969) daily inflows of the Tanghe Reservoir were used as historical time series. The averages and standard deviations of daily inflows were obtained and reflected in Figure 3. It can be seen that the average values was roughly independent and uniformly distributed. The inflows of the Tanghe Reservoir were extremely uneven during the year, and the inflow in July and August accounted for 49.4% of the annual inflow. The high flow only lasted for a short period of time.

**Figure 4:**
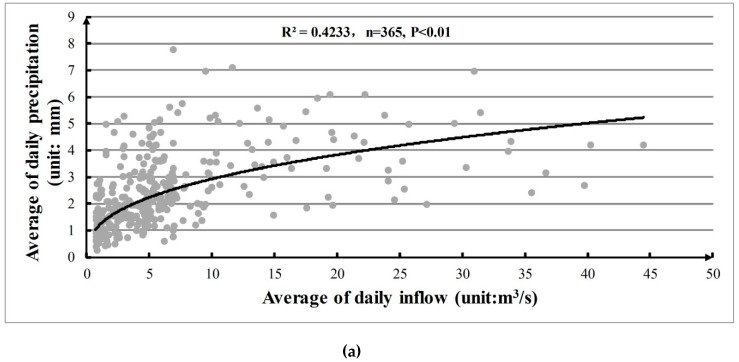

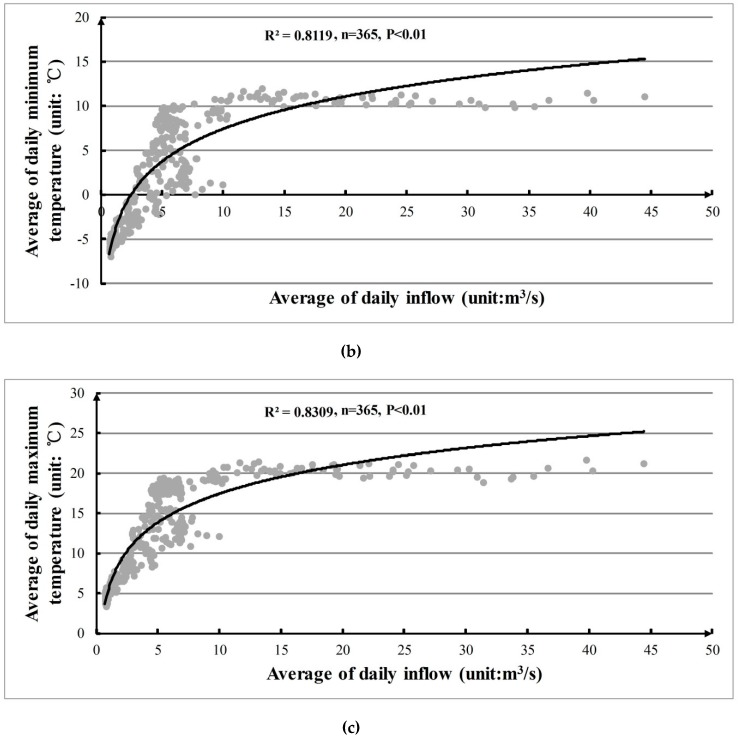
Dependence of the average of daily inflow on average of daily precipitation, minimum temperature, and maximum temperature. (**a**) Relationship between average of daily inflow and precipitation; (**b**) Relationship between average of daily inflow and minimum temperature; (**c**) Relationship between average of daily inflow and maximum temperature.

The daily precipitation, minimum temperature, and maximum temperature from 1950 to 1969 at the Tanghe Gauging Station were also obtained for the statistical analysis for hydroclimatology parameters. We plotted the dependence of average of daily inflow on average of daily precipitation, minimum temperature and maximum temperature shown in Figure 4. The averages of daily inflow generally increase with the average of daily precipitation, minimum temperature and maximum temperature. Compared with Figure 4a–c, it can be found that the inflows are more convincingly related to the minimum temperature (*R*^2^ = 0.8119, *p* < 0.01) and maximum temperature (*R*^2^ = 0.8309, *p* < 0.01). This indicates that the inflow of reservoir is not just water transformation from precipitation but also is strongly influenced by the temperature variation. This information proved that the inflows of reservoir could be influenced by the climate change. Thus, directly using the historical inflows without the consideration of uncertainty in inflow will lead to poor performance of reservoir operation. 

### 4.2. Performance Tests for Monte Carlo GA and NGA

To test the performance and sensitivity of the Monte Carlo GA and the NGA stochastic models, we used Policy 3 as an example. In the Monte Carlo GA, inflow is generated based on the average and standard deviation values of historical inflow data shown in Figure 3. The method for daily inflow generation is similar to the Monte Carlo method [45]. To fully consider the stochastic nature of daily inflows, the Monte Carlo GA needs a great deal of realizations to get the final solution for a noisy environment. In accordance with prior research, 1000 realizations were used [24]. In the NGA, the inputs are historical data and the stochastic nature of inflow is solved by using the sampling fitness function to reduce the noise of fitness assessment. According to the parameters given in Table 3, the Monte Carlo GA and the NGA optimize reservoir operation independently. Equation (4) was explored as the objective function targeting maximum water supply reliability and parameters *α* and *β* were assumed to equal 20 and 30, respectively [11,35,39]. MATLAB 7.1was used to complete the reservoir optimal operation process. 

#### 4.2.1. Parameter Setting for the Monte Carlo GA

**Figure 5:**
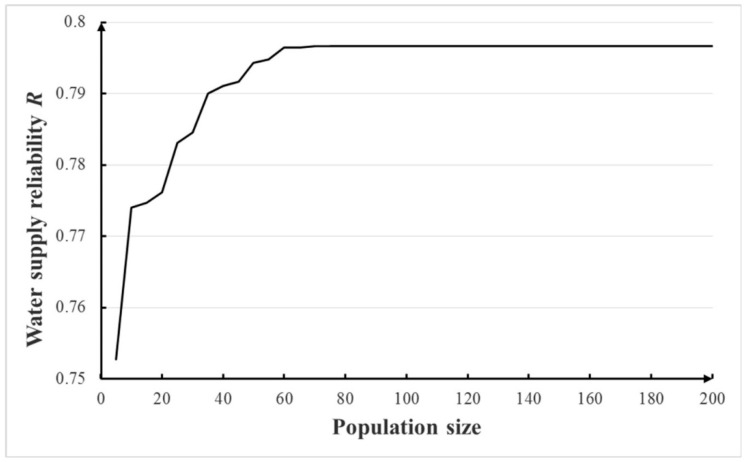
Sensitivity of population size in Monte Carlo GA.

**Figure 6:**
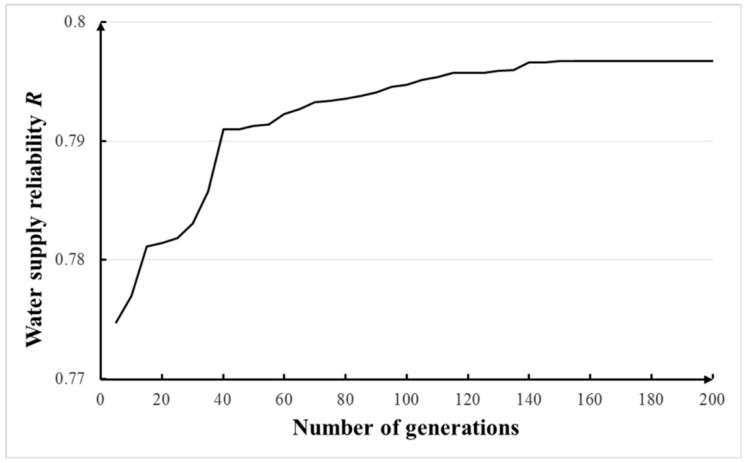
Sensitivity of number of generations in Monte Carlo GA.

In this section, with the other parameters listed in Table 3, the population size and number of generations of Monte Carlo GA were tested. The results of total 1000 realizations based on the Monte Carlo method were averaged as the final solution. The sensitivities of population sizes of 5–200 and number of generations of 5–200 were tested and the results are shown in Figure 5 and Figure 6. Based on these figures, the population size of 70 and the number of generations of 150 were adopted in this study. For the Monte Carlo GA, the computational time of each optimization cost ~55 min and 30 s on the computer with 2 GB RAM memory and a 2.0 GHz CPU. 

#### 4.2.2. Parameter Setting for the NGA

**Figure 7:**
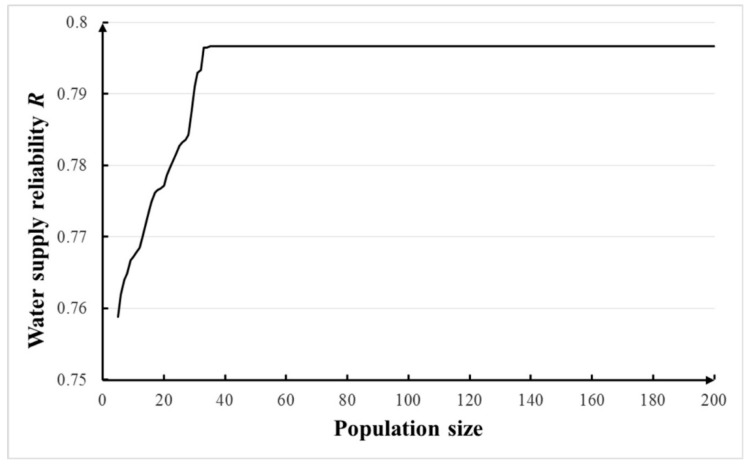
Sensitivity of population size in noisy GA.

**Figure 8:**
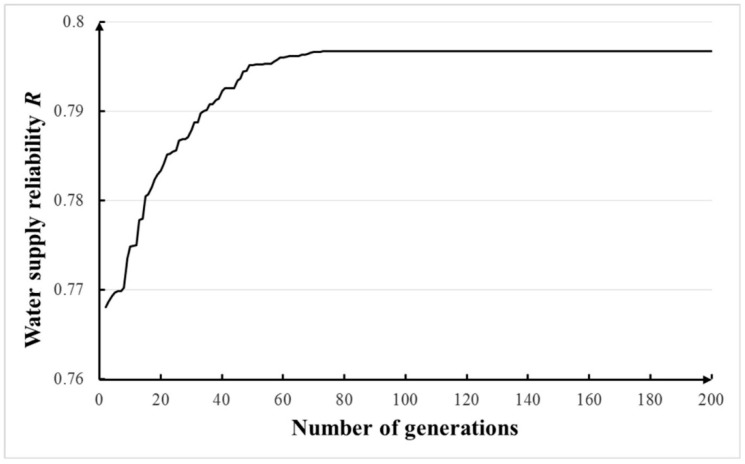
Sensitivity of number of generations in noisy GA.

With other parameters shown in Table 3, sensitivities of population sizes of 5–200 and number of generations of 5–200 were tested and the results are shown in Figure 4. It has been proved that larger sample sizes do not guarantee better optimal results. For simplicity, a sample size of five was chose according to Branke [44]. Seen in Figure 7 and Figure 8, it can be concluded that the population size of 40 and number of generations of 80 can be applied in NGA. The calculation of each optimization costed ~5 min and 40 s on the computer with 2 GB RAM memory and a 2.0 GHz CPU. 

### 4.3. Comparison of the Two Stochastic Models

To test the effectiveness of the Monte Carlo GA and NGA, we considered two different stochastic inflow conditions for the optimization of ecofriendly reservoir operation. The first condition considered the stochastic inflow in a traditional way based on the average and standard deviation of historical inflows. The second condition is based on the changed average (±10%) and standard deviation (±10%) of historical inflows. With five inflow conditions, two stochastic optimization models, and 18 release policies, 5 × 2 × 18 sets of optimal RORCs were finally achieved. 

#### 4.3.1. Optimization Based on the Average and Standard Deviation of Historical Inflows 

Regarding the Monte Carlo GA, *n* realizations of daily inflows were generated based on the Monte Carlo method using the daily average and standard deviations of daily inflows are shown in Figure 3. Each generated realization was used as the input of ecofriendly reservoir operation and the GA was adopted to optimize the operation. Then the multiple optimal solutions were average as the final decision. To fully consider the stochastic character of daily inflow, 1000 realizations were used [33]. As for the NGA, twenty years of daily historical inflows were used as the input. During the sampling process, the average and standard deviation of daily inflows were used as the bound to adopt range of disturbances for the expected fitness function.

(1) Water supply reliability (*R*) 

**Figure 9:**
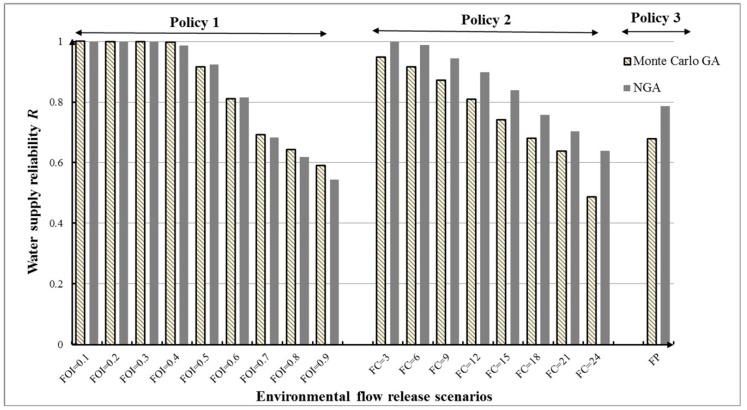
Water supply reliability *R* for different e-flow release policies under different models.

Figure 9 and Table 4 show the maximum value of water supply reliability under three different e-flow release policies (18 scenarios) for the two optimization models. Water supply reliability, *R*, is clearly different under different models. It can be seen that the obtained water supply reliability *R* of NGA is clearly higher than that of Monte Carlo GA, aside from four scenarios (FC = 0.4, 0.7, 0.8, 0.9) where the results of the two methods are relatively similar. Compared to the Monte Carlo GA, NGA recognized 78% cost-effective solutions without extensive computational effort. Under the same e-flow release policies, the NGA could perform better than the Monte Carlo GA in stochastic ecofriendly reservoir operation. Although e-flow allocation can decrease the water supply reliability in reservoir operation, using the NGA to optimize reservoir operation could ensure higher water supply reliability under the same conditions. 

**Figure 10:**
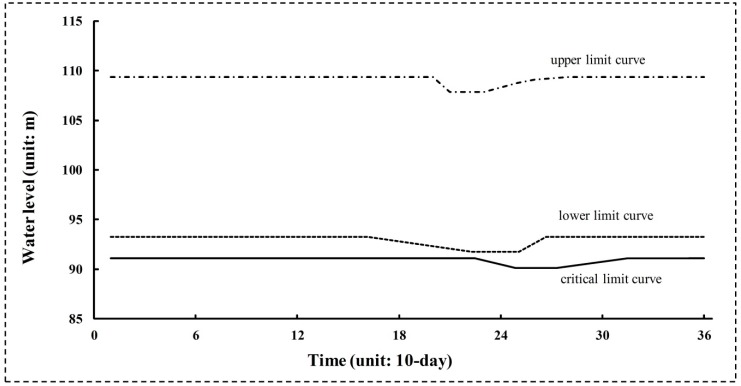
Optimal reservoir operating rule curves (RORCs) with 100% water supply reliability under the FOI = 0.1 scenario by NGA.

Under this inflow condition with 18 release policies, 18 × 2 = 36 sets of optimal RORCs were finally achieved. The optimal RORCs with 100% water supply reliability under FOI = 0.1 scenario optimized by NGA were plotted as in Figure 10. According to the rules in Figure 10, the reservoir managers could completely satisfy water supply reliability for human. 

(2) Flow regime alteration (*D*) 

**Figure 11:**
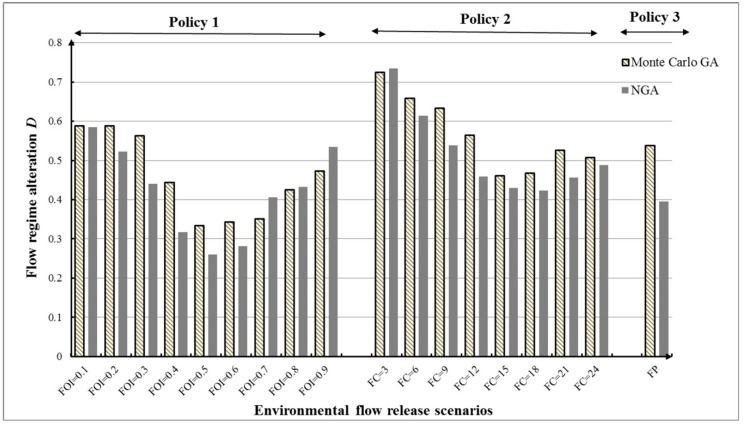
Flow regime alteration *D* for different e-flow release policies under different models.

Figure 11 and Table 5 illustrate total flow regime alteration (*D*) corresponding to three e-flow release policies under the first inflow condition. We can see that although minimizing flow regime alteration *D* is not our objective, most values of *D* (~83%, except scenarios FOI = 0.7, 0.9, and FC = 3) obtained by the NGA are lower than those based on the Monte Carlo GA. The lower degree of flow regime alteration means that the downstream ecosystem could obtain better protection. The NGA can identify lower *D* for riverine ecosystem and higher *R* for human interest at the same time, which is proved to be effective tool for ecofriendly reservoir operation under uncertainty.

**Figure 12:**
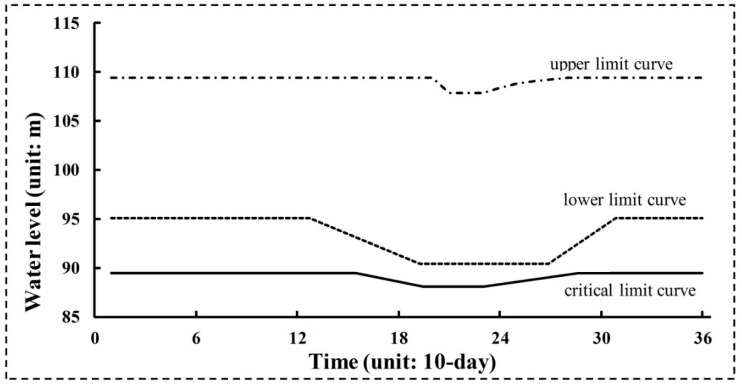
Optimal reservoir operating rule curves (RORCs) with 0.3958 flow regime alteration under FP scenario optimized by NGA.

Meanwhile, a comparison of different values of *D* in Figure 11 indicates that total flow regime alteration does not always decrease as e-flow increases. In Policy 1, it can be seen that the variability in *D* of the results shows a U-form. For values less than FOI = 0.6 (fraction of inflow for e-flow), the *D* decreased as fraction of inflow for e-flow increased. For values greater than FOI = 0.6, *D* presents a growth trend. This demonstrates that *D* is more sensitive than *R* in e-flow policies. The optimal RORCs with 0.3958 flow regime alteration under FP scenario optimized by NGA were plotted as in Figure 12. According to the rules in Figure 12, the reservoir managers could well protect the downstream flow regime for riverine ecosystem.

(3) Finding the final solution

**Figure 13:**
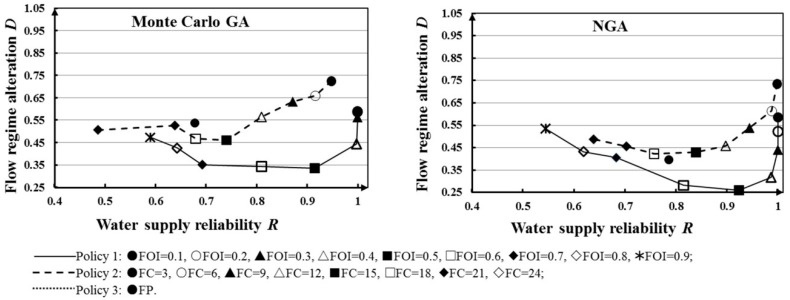
Illustration of the tradeoff between the total flow regime alteration R and water supply reliability, D, based on Monte Carlo GA and NGA.

To identify the final solution with low *D* and high *R*, we have combined the results for each scenario under different optimization methods as shown Figure 13. For the 18 scenarios, Figure 9 compares the total *D* and *R* under the Monte Carlo GA and the NGA. The results show that reservoir operation with e-flow release scenario FOI = 0.5 could obtain higher water supply reliability and lower flow regime alteration than other scenarios under the two methods. Meanwhile, the NGA performs well in reservoir operation under this kind of inflow condition. 

#### 4.3.2. Optimization Based on Changed Average and Standard Deviation 

In this section, the average and standard deviation of historical inflow shown in Figure 3 were designed to be changed with ±10%, respectively. Those conditions were designed to compare performance of the NGA and the Monte Carlo GA under a range of stochastic conditions. The Monte Carlo method was used to generate 1000 realizations based on the changed average and standard deviation of daily inflow. The simple GA used the each realization as input to optimize the reservoir operation, and the results were averaged as the final solution. NGA also used the historical inflow as input, while the changed average and standard deviation as the bound of disturbances. 

**Figure 14:**
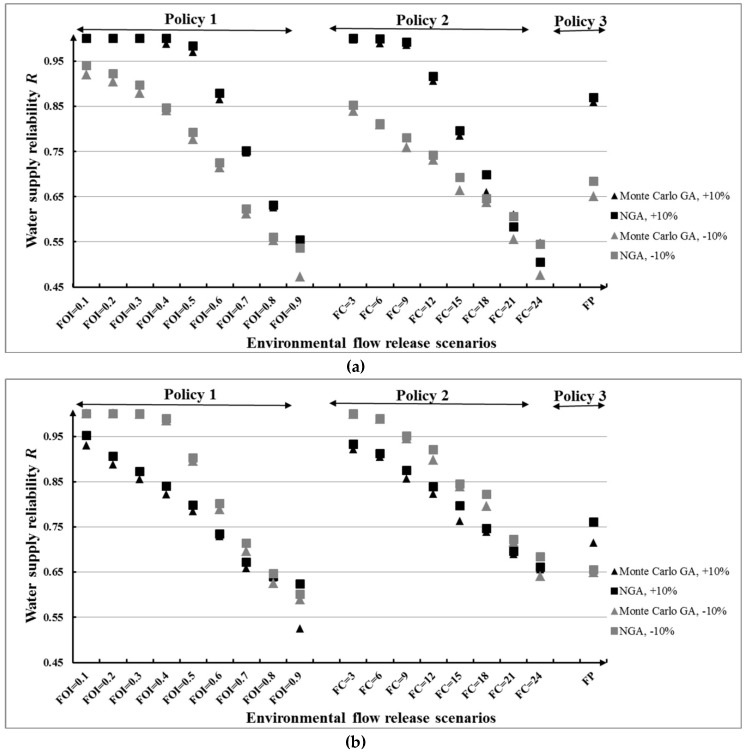
Water supply reliability *R* for different e-flow release policies under different inflow conditions: (**a**) Change of average value and (**b**) change of standard deviation value.

Figure 14a,b illustrates water supply reliability *R* for the three e-flow release policies under the second stochastic inflow condition. It is evident that *R* is influenced by changes in inflow for all policies. As expected, the NGA produced higher *D* values than the Monte Carlo GA. It can be seen that in Figure 14a almost all values of *R* under the increase of inflow average are higher than that under the decrease of inflow average. This is easy to be understood. The increase of inflow average means an increase in the total amount of water in river, leading to greater satisfaction of water supply requirement of human and higher *R*. In contrast, in Figure 14b, the values of *R* under the increase of inflow standard deviation are lower than that under the decrease of inflow standard deviation. That is to say, when the standard deviation of daily inflow decreased, the inflows are slightly different from each other and it will easier to obtain higher *R*. A comparison of the four changed inflow conditions in Figure 14 indicates that high average or low standard deviations of inflows correspond with higher *R*. 

**Figure 15:**
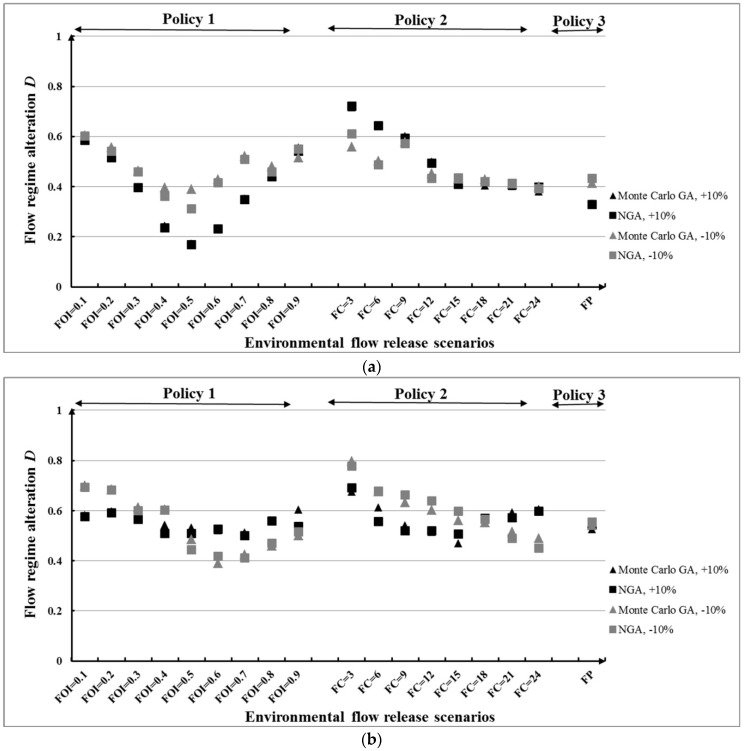
*D* for different e-flow release policies under different inflow conditions: (**a**) Change of average value and (**b**) change of standard deviation value.

Figure 15 illustrates the flow regime alteration *D* for the three e-flow release policies under the second inflow condition based on Monte Carlo GA and NGA. It is evident that *D* is influenced by the changes in inflows for all policies. In most cases, the NGA produced lower *D* values than the Monte Carlo GA. We can see in Figure 15a that the values of *D* under the increase of inflow average are not always lower than that under the decrease of inflow average. This is to say, an increase in the total amount of water in river does not mean the river downstream will obtain better protection. This kind of protection much depends on the e-flow release scenarios. It is obvious in scenarios FOI = 0.8, FOI = 0.9, FC = 18, FC = 21, and FC = 24, where the *R* is almost the same with different inflow condition and optimization methods. Thus, the choice of e-flow release scenario is also an important way to influence the performance of reservoir operation.

In Figure 15b, the results of *D* under the conditions of increase and decrease of inflow standard deviation in policy 1 and policy 2 crossed with each other. In scenarios FOI = 0.1 (FC = 3) to FOI = 0.4 (FC = 15), the values of *R* under the increase inflow standard deviation are lower than that under the decrease condition, which is in contrast with scenarios FOI = 0.5 (FC = 18) to FOI = 0.9 (FC = 24). A comparison of the four changed inflow conditions in Figure 15 indicates that, high average inflows correspond with lower *D*, while the influence of standard deviation on *D* is depends on the choice of e-flow scenario.

In general, based on these results, we conclude that, under different stochastic inflow conditions and e-flow release scenarios, NGA could identify more reliable and cost-effective solutions with about 90% less computational effort than based on Monte Carlo GA. Meanwhile, the optimization with e-flow scenario FOI = 0.6 often obtained a lower flow regime, which can be recommended as the candidate solution for reservoir operation. 

## 5. Conclusions

In this study, we developed a Monte Carlo GA and a NGA for ecofriendly reservoir operation optimization, taking the stochastic nature of inflows into consideration. Using the Tanghe Reservoir in China as an example, the results of this study showed that a NGA can be a useful tool for ecofriendly reservoir operation under stochastic inflow conditions.

In solving the problem, for Monte Carlo GA, a classical simple of a GA, needs to repeatedly used to research the final solution. Instead of redundant repeated computation, a noisy GA could just run once and get the optimal solution, simplifying the optimization process.

(1) A NGA could greatly reduce the computational time for optimization over that of a Monte Carlo GA, which needs to be used repeatedly for every realization. The NGA could find an optimal solution the first time based on the sampling fitness function. The NGA will lead to lower flow regime alteration and higher water supply reliability under some e-flow release policies.

(2) This study suggests that the NGA is better than the Monte Carlo GA for stochastic ecofriendly reservoir operation. However, the NGA may not be a better alternative than all other optimizing methods. We have only used a single water supply reservoir as an example. The effectiveness of the NGA in other reservoirs or systems remains a topic for further study.

## Figures and Tables

**Table 1:** Detailed information of the different environmental flow policies and related scenarios.

Policy	Scenarios	Hypothesized Conditions
1. Fraction of Inflow (FOI)	Scenario 1, FOI = 0.1; Scenario 2, FOI = 0.2; ⁞ Scenario 9, FOI = 0.9.	This policy offers a promising way that allows for extensive trade-offs between water supply for human and environmental flow requirement.
2. Flow Components (FC)	Scenario 1, 10% of ADF as e-flow during dry season and 30% during the wet season, and 3*1 high-flow events; Scenario 2, 10% of ADF as e-flow during dry season and 30% during the wet season, and 3*2 high-flow events; ⁞ Scenario N (N = INT[the total number of high-flow events/3]), 10% of ADF as e-flow during dry season, 30% during the wet season, and 3*N high-flow events.	This policy attempts to provide occasional high-flow releases for habitat improvement.
3. Four-period release approach (FP)	Scenario 1, (i) Floods: bankfull discharge as e-flow; (ii) Base flows: 10% of ADF as e-flow during the dry season and 30% of ADF during the wet season; (iii) Extreme low flows: e-flow equals to the reservoir inflow; (iv) High-flow pulses: all high-flow are released.	This policy attempts to provide the full range of natural flow regime alterations, including floods and droughts, in which the riverine ecosystem is adapted.

**Table 2:** Indicators of hydrological alteration (IHA) in the range of variability approach.

IHA Group	Hydrological Indicators
Group 1: Magnitude of monthly water conditions	Mean value for each calendar month.
Group 2: Magnitude and duration of annual extreme water conditions	Annual minima 1-day means; Annual maxima 1-day means; Annual minima 3-day means; Annual maxima 3-day means; Annual minima 7-day means; Annual maxima 7-day means; Annual minima 30-day means; Annual maxima 30-day means; Annual minima 90-day means; Annual maxima 90-day means.
Group 3: Timing of annual extreme water conditions	Julian date of each annual 1 day maximum; Julian date of each annual l day minimum.
Group 4: Frequency and duration of high and low pulses	No. of high pulses each year; No. of low pulses each year; Mean duration of high pulses within each year; Mean duration of low pulses within each year.
Group 5: Rate and frequency of water condition changes	Means of all positive differences between consecutive daily means; Means of all negative differences between consecutive daily values; Number of rises; Number of falls.

**Table 3:** Components and initial parameters of the Monte Carlo Genetic Algorithm (GA) and the Noisy Genetic Algorithm (NGA).

Component and Parameter	Type and Value	Component and Parameter	Type and Value
Representation	Real	Crossover	Scattered, 0.7
Selection	Tournament, 4	Mutation	Uniform, 0.08

**Table 4:** Water supply reliability *R* for Monte Carlo GA and NGA models.

Policy		Stochastic Model	
		Monte Carlo GA	NGA
1	FOI = 0.1	1	1
	FOI = 0.2	0.9996	1
	FOI = 0.3	0.9995	1
	FOI = 0.4	0.9976	0.9867
	FOI = 0.5	0.9151	0.9237
	FOI = 0.6	0.8099	0.8148
	FOI = 0.7	0.6925	0.683
	FOI = 0.8	0.6425	0.619
	FOI = 0.9	0.5895	0.5447
2	FC = 3	0.9479	0.9987
	FC = 6	0.9162	0.9879
	FC = 9	0.8713	0.9443
	FC = 12	0.8096	0.8977
	FC = 15	0.7405	0.8395
	FC = 18	0.6795	0.7574
	FC = 21	0.6382	0.703
	FC = 24	0.4862	0.6386
3	FP	0.6778	0.7863

Note: FOI, FC, and FP represent fraction of inflow, flow components, and four-period release approach, respectively.

**Table 5:** Flow regime alteration *D* for the Monte Carlo GA and the NGA.

Policy		Stochastic Model	
		Monte Carlo GA	NGA
1	FOI = 0.1	0.5875	0.5854
	FOI = 0.2	0.5875	0.5229
	FOI = 0.3	0.5625	0.4403
	FOI = 0.4	0.4438	0.3174
	FOI = 0.5	0.3344	0.2597
	FOI = 0.6	0.3431	0.2813
	FOI = 0.7	0.3513	0.4056
	FOI = 0.8	0.4250	0.4326
	FOI = 0.9	0.4731	0.5347
2	FC = 3	0.7250	0.7347
	FC = 6	0.6588	0.6139
	FC = 9	0.6325	0.5382
	FC = 12	0.5644	0.4583
	FC = 15	0.4606	0.4292
	FC = 18	0.4669	0.4229
	FC = 21	0.5263	0.4563
	FC = 24	0.5069	0.4875
3	FP	0.5375	0.3958

Note: FOI, FC, and FP represent fraction of inflow, flow components, and four-period release approach, respectively.
